# A study of the influence of current ramp rate on the performance of polymer electrolyte membrane fuel cell

**DOI:** 10.1038/s41598-022-25037-0

**Published:** 2022-12-19

**Authors:** Mathan Chandran, Karthikeyan Palaniswamy, N. B. Karthik Babu, Oisik Das

**Affiliations:** 1 Fuel Cell Energy System Lab, Department of Automobile Engineering, PSG College of Technology, Coimbatore, Tamilnadu India; 2 Department of Automobile Engineering, PSG College of Technology, Coimbatore, Tamilnadu India; 3 Department of Mechanical Engineering, Assam Energy Institute, Centre of Rajiv Gandhi Institute of Petroleum Technology, Sivasagar, Assam 785697 India; 4 Structural and Fire Engineering Division, Department of Civil, Environmental and Natural Resources Engineering, Luleå University of Technology, 97187 Luleå, Sweden

**Keywords:** Fuel cells, Engineering

## Abstract

Durability and reliability are the key factors that prevent fuel cells from successful implementation in automotive sector. Dynamic load change is a common and frequent condition that the fuel cell has to undergo in automotive applications. Fuel cells are more sensitive to changes in load conditions and degrade based on load variation representing idling, rated power, and high power operating conditions. To examine the influence of dynamic load step on the fuel cell performance, two similar cells of active 25 cm^2^ was tested under two different load step for the same dynamic load cycle. The main difference in dynamic load cycle 2 was the ramp rate which was fixed as 0.1, 0.3, and 0.25 A/cm^2^/s for 0.2, 0.6, and 1.0 A/cm^2^ respectively. To investigate the degradative effects, polarization curves, electrochemical impedance spectroscopy, and field emission scanning electron microscopy were used. The results indicated that the degradation rate increased in both dynamic load cycles but however the impact of load change was comparatively minimal in dynamic load cycle 2. The total degradation in performance was 20.67% and 10.72% in dynamic load cycles 1 and 2 respectively. Fuel cell performance degraded in a manner that was consistent with the electrochemical impedance spectroscopy and cross-sectional analysis of field emission scanning electron microscopy. The results prove that the degradation rate is dependent on the load step and the number of load cycles. Severe catalyst degradation and delamination were observed in fuel cells operated under dynamic load cycle 1.

## Introduction

Polymer electrolyte membrane fuel cell/Proton exchange membrane fuel cell (PEMFC) is one of the likely clean energy generating solutions for automotive, special vehicles, and stationary applications. PEMFCs are under the spotlight due to their high efficiency compared to other energy conversion devices, power and current density, relatively faster start-up, and zero-emission^1^. PEMFCs as vehicle powering source has been matured due to immense research globally and is evident by the successful operation of Hyundai IX35 and Toyota Mirai^2^. Despite many research advancements, fuel cells have hindrances in commercialization. This is due to higher cost as platinum is used as a catalyst, shorter lifetime, reliability, and lack of infrastructure^3,4^. PEMFC lifetime in the automotive application as per the US Department of Energy is 8000 h with only 10% loss in performance^5^.

**Figure 1:**
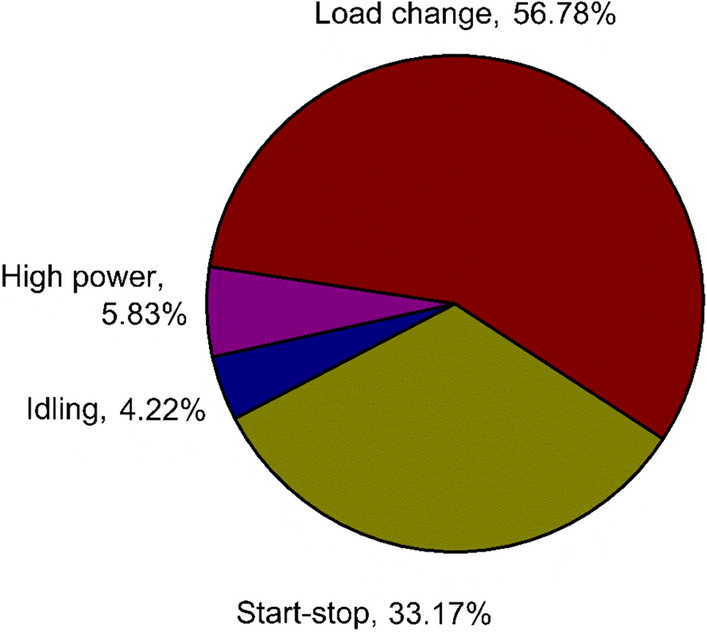
Degradation percentages of different operating conditions in a PEMFC.

Various factors that affect the lifetime of fuel cells in real-time application are pollution^6,7^, gas starvation, and local cell reversal^8^. During real-time operation, a fuel cell operates at different load conditions like start-stop^9–11^, idling^12–15^, and high power^16^. The percentage of degradation contribution by each operating condition is shown in Fig. 1. From Fig. 1, it can be identified that the load change is one of the major influential factor for performance degradation contributing up to 56.78% and the second major factor is the start-stop operation contributing a total of 33.17%. The detailed discussion about the different contributing factors are explained in^17^. It’s essential to contemplate the impact of each load condition while evaluating the PEMFC’s performance degradation.

Start–stop is an inevitable and frequent process in automotive applications. During the start/stop process hydrogen-air boundary may be formed at the anode and leads to carbon corrosion due to the high interfacial potential difference between anode and cathode (can be as high as 1.5 V)^18–21^. This carbon corrosion weakens the entire catalyst structure and increases the charge and mass transfer resistance^22–24^. Idling is more frequent in urban operating conditions. Idling indicates that the fuel cell supplies power demanded by the subsystem like water pump, supercharger, hydrogen injector, anode recirculation blower, and other electrical systems. Usually, the total power demand by all these subsystems is not more than 1% of the rated fuel cell power not considering the compressor power consumption which usually accounts for nearly 15–20%^14,25^. In this work compressor power is neglected as O_2_ is directly utilized in this experiment. In a fuel cell working under idling conditions, the cathode potential might be higher than 0.8 V. During this operation, carbon corrosion on the cathode occurs readily and accelerates the Pt agglomeration and dissolution^26^. In addition, chemical degradation of the membrane occurs due to reacting with hydrogen peroxide and free radicals formed during the electrochemical reaction^23,27^.

Fuel cell needs to operate under high power condition for a very short duration while accelerating, and hill climbing. During these operation, fuel cell undergoes reactant starvation, local hot spot formation, and flooding due to high current production^28,29^. This lead to membrane chemical degradation, carbon corrosion, Pt agglomeration, and Pt dissolution. Another operating condition the fuel cell has to undergo during the actual driving is the frequent change of load. This condition is the major factor that affects the fuel cell performance degradation the most^30^. During load changing the fuel cell potential changes and the electrochemical products like water and heat also changes according to the load. Eventually, an internal cycling of humidity and/or temperature takes place which in turn accelerates the chemical and mechanical degradation of the components^31^.

Actual on-road performance and durability tests on PEMFCs are time-consuming, high cost and involvement of multi-factor effect, fuel cell test protocols based on drive cycles were developed^32^. New European Drive Cycle (NEDC) is the basis of the fuel cell lifetime cycle test developed by the International Electrotechnical Commission (IEC), where the actual load demand by the vehicle is converted into current–time condition for a fuel cell system.

Borup and Mukundan experimentally investigated the PEMFC degradation using a modified drive cycle. From their results, the catalyst size growth depends on the operating current density^33^. Zhao et al. investigated the dynamic behaviour of a 2 kW PEMFC stack under dynamic varying loads. The results revealed that when the external load increased, the oscillation rate and dynamic resistance reduced more rapidly^34^. Durst et al. studied the formation of hydrogen-air boundary formation during the start-up of the fuel cell. Interestingly they found that regions, where there was hydrogen starvation acted as electrolyser drawing current from the cell and the hydrogen-rich region acted as a normal fuel cell^35^. The effect of varying flow rates during start-up and shut-down was investigated by Brightman et al. for flow rates of 200, 600, and 1000 ml/min. It was concluded that the carbon corrosion decreased as the flow rate increased. This was due to the shorter residence time of the gases present inside the fuel cell^36^. Cheng et al. studied the voltage decay rate of a stack for 4000 h operated under 100% humidification, ambient pressure, and H_2_ and O_2_ conditions. They reported that the voltage degraded about 3.1 µV/h at 0.4 A/cm^2^ current density^37^. Mocoteguy et al. evaluated the electrochemical and dynamic characteristics of a 500 W PEMFC stack. They concluded that the individual cell performance decreased rapidly at the end of the aging test as the ohmic resistance of the cells increased^38^. Kakizawa et al. evaluated the effect of the partial pressure changes of oxygen with 3, 5, and 10% oxygen in air and found that, the oxygen starvation lead to the hydrogen evolution reaction in the cathode and this in turn reduced the overall performance. In addition the performance degradation was supported by the reduction of electrochemical active surface area which reduced from 69.9 to 67.6 m^2^/g and oxygen consumption by the hydrogen evolved on the cathode was found to be 0.07–0.7%^39^.

Though there are a lot of researches that have analysed the effect of drive cycle on the PEMFC performance degradation, they lack in emphasising the combined effects of operating parameters and load ranges. In this study, the effect of the load step of the dynamic load cycle is experimentally evaluated. The primary motive of the study is to determine the effect of the intermediate load step when varying the load from one current density to another current density. This work exhibits the different performance degradation rates of the fuel cell at idling, rated, and high load conditions. Degradation behaviour was investigated periodically during the dynamic load cycle by conducting polarization curve, electrochemical impedance spectroscopy (EIS), and finally by field emission scanning electron microscopy (FESEM) to investigate the microscopic degradation of the catalyst, catalyst support, and membrane. From the results of this paper, different degradation mechanisms can be investigated in depth and a suitable control strategy can be developed to mitigate these effects in real-time operating conditions.

## Experimental

### Test bench

Biologic FCT-50S was used as a test bench for the dynamic load cycle test; it includes the humidifier, reactant supply system, control system, safety system, and data acquisition system. The power capacity of the test bench is 250 W. The exhaust hydrogen is safely vented out by the external exhaust system. The test bench was specially designed for low temperature, low power fuel cell application and has accurate humidification, reactant distribution, and pressure and temperature control for both anode and cathode.

### Test cell and test conditions

A gas diffusion electrode (GDE) with a Pt loading of 0.5 mg/cm^2^ 40% Pt/C with a thickness of 200 µm was used and the membrane used was Nafion 115 with a thickness of 124 µm. The membrane electrode assembly (MEA) was prepared by inserting the membrane between anode and cathode electrodes in a hydraulic hot press under 25 kg/cm^2^ pressure and 130 ℃. A serpentine flow channel was employed on both anode and cathode flow plates. The physical specification of the single cell used for testing are given in Table 1.

**Table 1:** Geometric parameters of the tested cell.

Parameter	Value
Active area (m^2^)	25 × 10^–4^
Flow field width (m)	2 × 10^–3^
Flow field depth (m)	2 × 10^–3^
Channel rib width (m)	2 × 10^–3^
Membrane thickness (m)	1.24 × 10^–5^

Mylar sheet was used as a gasket to prevent reactant leakage on either side of the membrane. The test cell was assembled and fastened by 8 bolts with a torque of 11 Nm. After the cell was assembled and checked for leakage. Before the start of the dynamic load cycle test, the MEA needs to be activated for proper hydration of the membrane. The MEA was activated by the current density and duration as follows 0.02, 0.05, 0.10, 0.20, 0.30, 0.40, 0.50, 0.60, 0.70, 0.80, 0.9, and 1.0 A/cm^2^ for 300 s each and 0.70 and 0.80 for 3600 s each followed by maintaining open circuit voltage (OCV) for 60 s this procedure was repeated for 5 cycles before the actual test. Operating parameters are given in the Table 2. Throughout the experiment the cell temperature was maintained at 60 °C and the reactant flow rate was 270 ml/min and 169 ml/min for H_2_ and O_2_ respectively. The activation procedure is repeated 5 times and the total duration was about 15 h.

**Table 2:** Operating parameters for polarization curves.

Inlet stoichiometry		Relative humidity (%)		Inlet pressure (kPa)	
Anode	Cathode	Anode	Cathode	Anode	Cathode
1.2–7.2	1.5–8.9	100	70	120	100

### Polarization curves and dynamic load test

The fuel cell performance was recorded using the polarization curve, before, during, and after the dynamic load cycle test. The fluctuations in transient voltage that occur throughout each driving cycle cannot be used to demonstrate fuel cell degradation. So, it is necessary to record the polarization curve of the cell to evaluate the performance degradation. Polarization curves were measured for every 100 cycles of the dynamic load test. Based on the current density, the stoichiometry ratio of anode and cathode varies from 1.2–7.2 and 1.5–8.9 respectively.

**Figure 2:**
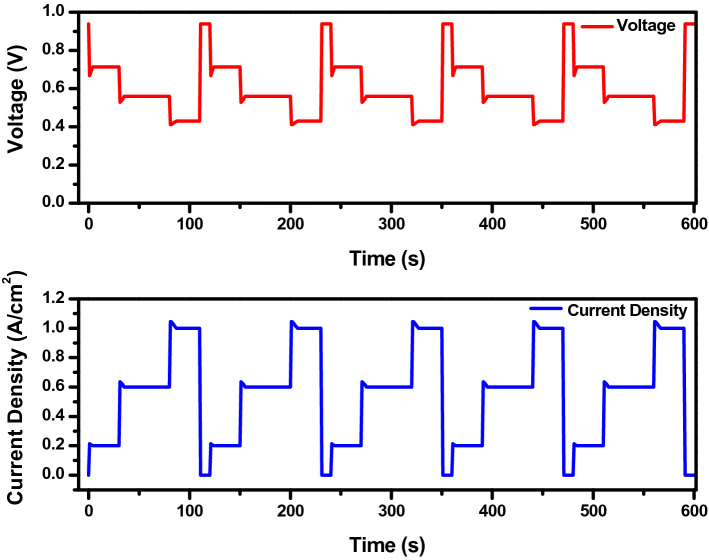
Dynamic load cycle voltage and current profile.

The fuel cell was tested for its durability under dynamic load conditions, developed by modifying the load demand by the vehicle operating with an internal combustion engine considering operations like idling, rated power, and high power conditions to the current–time profile suitable for fuel cell testing. In this study, to include the effects of start-stop operating conditions, the cell was maintained at OCV condition at the end of each dynamic load cycle for 10 s. Two dynamic load cycles were developed for this study. The load profile of dynamic load cycle 1 (DLC1) and dynamic load cycle 2 (DLC2) are given in Table 3. The dynamic load cycle investigation was carried out for 2000 cycles for each DLC or till the performance drop was equal to or higher than 10% of the initial performance before the start of the dynamic load cycle test. Voltage and current for each cycle were recorded and for reference some cycle’s voltage and current profile is presented in Fig. 2. Throughout the test, the cell temperature was maintained at 60 ℃ and relative humidity was 100% and 70% for anode and cathode in both DLC tests. The loading time between two consecutive current densities was 1 s in both DLC. In DLC2 the ramp rate was 0.1 A/cm^2^/s, 0.3 A/cm^2^/s, and 0.25 A/cm^2^/s for 0.2 A/cm^2^, 0.6 A/cm^2^, and 1.0 A/cm^2^ respectively. After every 100 cycles, polarization curves, and EIS was measured and after the test, the MEA was cut and FESEM imaging was taken to analyse the morphology of the cross-section. In this work, 0.2, 0.6, and 1.0 A/cm^2^ was experimentally evaluated to test the combined influence of the idling, rated and high power operation of the stack corresponding to a vehicle’s operation. As described in Fig. 1, the load cycling has the major hold on the degradation of the fuel cell’s performance.

**Table 3:** Load profile of dynamic load cycle test.

Current (A)	Current density (A/cm^2^)	Duration/cycle (s)	Percentage of time (%)	Stoichiometry		Number of load steps	
				Anode	Cathode	DLC1	DLC2
5	0.2	30	25	7.2	8.9	1	2
15	0.6	50	42	2.4	2.9	1	2
25	1	30	25	1.4	1.8	1	4
0	0	10	8	–	–	–	–

### EIS test

The impedance test is one of the most powerful tests to clarify the degradation of PEMFC due to changes in MEA mechanical and chemical structure. To investigate the cell’s ohmic resistance, charge transfer resistance of oxygen reduction reaction (ORR), catalyst layer capacitance, and mass transfer resistance EIS was measured for every 100 cycles. EIS were carried out in potentiostatic mode under 0.5 V, the frequency range from 0.1 Hz to 1 kHz. Here, the high-frequency resistance characterizes the combined resistance of contact resistance between the components in a cell and ohmic resistance of the catalyst layer, membrane, gas diffusion layer (GDL), and flow plates. Low-frequency range represents the resistance due to mass transportation. The diameter of the semicircle denotes the charge transfer resistance in the cathode electrode and solid electrolyte interface, resistance due to the anode is not considered as it is negligible.

### FESEM analysis

Finally, after the dynamic load cycle was over, the MEAs were cut into pieces and subjected to FESEM imaging. This is done to analyze the effect of the dynamic load cycle on the structural changes of the catalyst and GDL, and the physical degradation of the carbon support and the membrane thickness. Any changes observed can be directly related to the change in EIS curves and can be confirmed as the reason for the performance degradation of the PEMFC. The effect of the dynamic load cycle’s loading steps on the performance degradation was focused on in this paper.

## Results and discussion

### Characteristics of dynamic loading cycle

**Figure 3:**
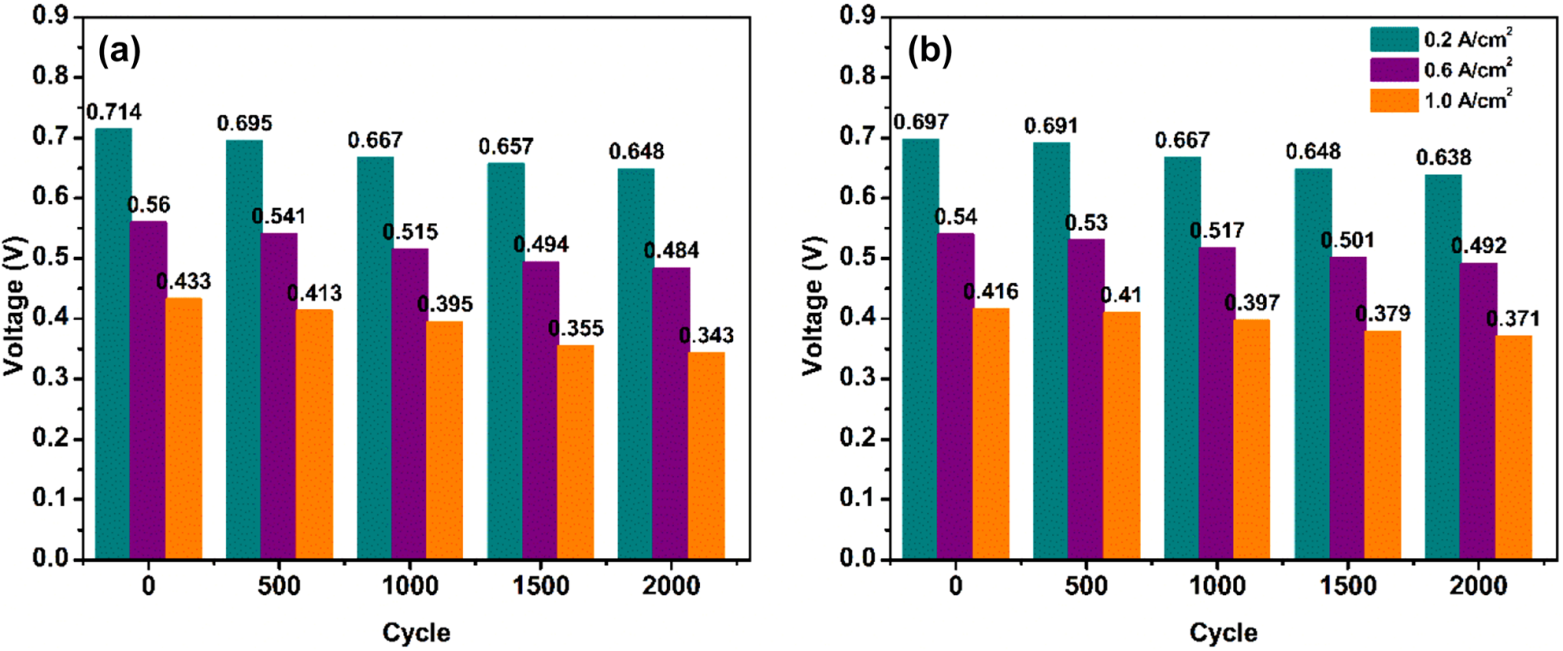
Voltage decay of (**a**) DLC1 and (**b**) DCL2 at 0.2, 0.6, and 1.0 A/cm^2^ current densities.

During loading or unloading, the voltage could rapidly change, undershoot and overshoot were observed instantaneously after the load change. This phenomenon got severe in DLC1 as the load changed rapidly, but was not that severe in DLC2 as the load was changed gradually. In both DLC1 and DLC2 the overshoot phenomenon increased as the cycles increased. However, in both cases, the cell recovered and attained a stable state. The initial drop in voltage during loading was a result of reactant starvation that occurs as a result of the mass transportation limitation of GDL and the fuel cell can’t cope up with the load changes like other energy conversion devices^40^. During unloading, there was a short-term spike in voltage as a result of excess reactants in the catalyst sites. The voltage degradation behavior of DLC1 and 2 is presented in Fig. 3. In DLC1, the voltage at 0.2 A/cm^2^ during the start of the test was 0.714 V, which decreased to 0.648 V after 2000 dynamic load cycles, accounts for a total voltage decay of 9.24%, while the voltage decrease was 13.57% at 0.6 A/cm^2^ and at 1.0 A/cm^2^ the voltage decreased from 0.433 to 0.343 V, corresponds to a total voltage decay of 20.78%. In DLC2, after 2000, at 0.2 A/cm^2^ the voltage decay was 8.46%, at 0.6 A/cm^2^ voltage decay observed was 8.88% and at 1.0 A/cm^2^ was 10.81%. In general, voltage decay increases as the number of cycles increases and the ramp rate increases. This phenomenon is more acute in higher current densities observed in DLC1, while in DLC2, the lower ramp rate is comparatively less severe.

During the change in current density, due to the mass transportation limitation of the GDL, short-term starvation of the reactants occurred inside the cell. Which ultimately leads to the performance degradation of the cell. In the cathode, when there is O_2_ starvation, the H^+^ ions that migrated to the cathode from the anode have less O_2_ than required. In this situation, H^+^ ions cannot take part in the ORR as a result, hydrogen evolution reaction (HER) takes place in the cathode^41,42^. Finally, cathode potential drops due to HER and can even lead to cell reversal. As a result of HER, the generated hydrogen present in the cathode can combine with the oxygen alongside the catalyst, and a considerable amount of heat was released. This heat caused local hotspots and can lead to catalyst sintering on the cathode side and on prolonged exposure to heat can develop pinholes on the membrane. In acute cases, the membrane can even burn out which results in the failure of the fuel cell.

In the case of H_2_ starvation, there are not enough H^+^ ions to continue the reaction. In this situation, to sustain the reaction, a carbon oxidation reaction and/or water electrolysis takes place to produce H^+^ ions. This drastically increases the anode potential and the output cell voltage decreases^43^. In broad-spectrum, a higher ramp rate results in reactant starvation which in turn increases the degradation rate of the catalyst layer and the membrane. Therefore, the fuel cell performance degradation increases.

### Analysis of polarization curve changes

**Figure 4:**
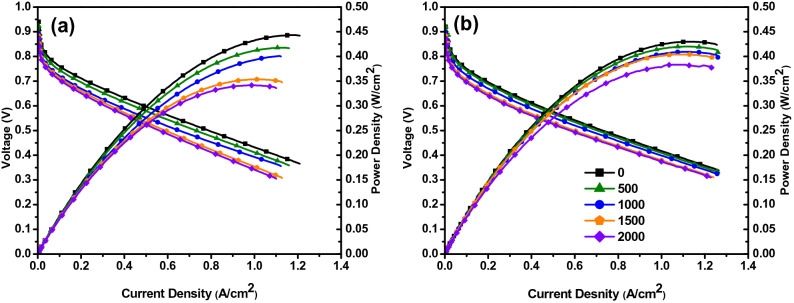
Polarization curves of (**a**) DLC1 and (**b**) DLC2 presented for every 500 cycles.

Polarization curves were measured every 100 dynamic load cycles. Polarization curves are a critical tool to identify the performance degradation of a fuel cell. Three different regions are observed in the polarization curve that corresponds to the activation region, ohmic region, and mass transportation region. It can visibly be seen that the performance decreases as the number of dynamic cycles increases. Another important observation made was that there was no apparent deviation in the lower current density operation, but as the current density increases, there was a significant deviation in the cell voltage^44^. The overall power of the fuel cell operated under DLC1 was decreased from 0.430 to 0.341 W/cm^2^, which accounts for a 20.581% total loss. Even though the performance has dropped well above the 10% performance drop mark, the experiment was continued for the sake of comparison of two load cycles and to attain the targeted 2000 cycles. While the fuel cell operated under DLC2 had a power drop from 0.429 to 0.385 W/cm^2^, accounting for 10.78% total loss. Polarization curves of fuel cells operated under DLC1 and DLC2 are shown in Fig. 4. From Fig. 4, it can be seen that the peak power voltage has reduced in both DLC1 and DLC2. The fuel cell performance degradation can be caused due to the carbon corrosion, of the Pt/C catalyst layer. It should be noted that the mechanisms of carbon corrosion in high and low current densities are different. During high current density, the water generated will be higher than the water removed from the cell, which causes flooding. Due to flooding, reactant starvation occurs at the catalyst site. This is one of the main causes of carbon corrosion. Another potential factor that accelerates the degradation is cell reversal due to reactant starvation^25^. Cell reversal has an adverse effect on GDL, catalyst layer, and even on the bipolar plate ^45^. When the cell is operated under high cell voltage—low current density, carbon corrosion can occur within a few hours of operation. This phenomenon is predominant in the cathode and follows the following reaction Eqs. (1, 2)^46^:

1 $$C+2{H}_{2}O\to {CO}_{2}+{4H}^{+}+{4e}^{-},\mathrm{ at }\, 0.207\,\mathrm{V}$$

2 $$C+{H}_{2}O\to CO+{2H}^{+}+{2e}^{-},\mathrm{at }\, 0.518\,\mathrm{V}$$

Carbon corrosion weakens the structure of the catalyst layer and ultimately leads to detachment and catalyst site isolation^47^. This can happen at high voltage conditions, such as 0.2 A/cm^2^ current density in this study. At the same time, fuel starvation due to water flooding at a high current density such as 1.0 A/cm^2^ in this study can also degrade the catalyst layer. When operating under starvation conditions, anode potential keeps rising and when the anode potential is finally higher than the cathode, it leads to cell reversal. During this situation, water electrolysis and carbon oxidation take place at the anode to supply the required amount of protons and electrons for a sustainable reaction. The reaction that takes place when the cell is flooded is different from that occurs at high cell potential. When the cell is flooded the reverse cell reaction follows Eq. (3):

3 $${H}_{2}O\to {}_{2}{}^{1}{O}_{2}+{2H}^{+}+{2e}^{-},\mathrm{ at } \,1.23\,\mathrm{V}$$

Power densities, total performance degradation and the degradation rate of DLC1 and DLC2 are tabulated in Table 4. The formula used for calculating the degradation rate is given in Eq. (4).

**Table 4:** Performance degradation of fuel cell operated under dynamic load conditions.

Load profile	Power density (W/cm^2^)		Power density degradation rate (µW/cm^2^ cycle)	Voltage (V)		Voltage degradation rate (µV/cycle)
	0th cycle	2000th cycle		0th cycle	2000th cycle	
DLC1	0.430	0.341	44.5	0.384	0.346	19
DLC2	0.429	0.383	23.0	0.352	0.331	10

4 $$Rate\, of\, Degradation=\frac{final\, power\, density\,(or\, voltage)-initial\, power\, density\,(or\, voltage)}{number\, of\, cycles}$$

### EIS analysis

**Figure 5:**
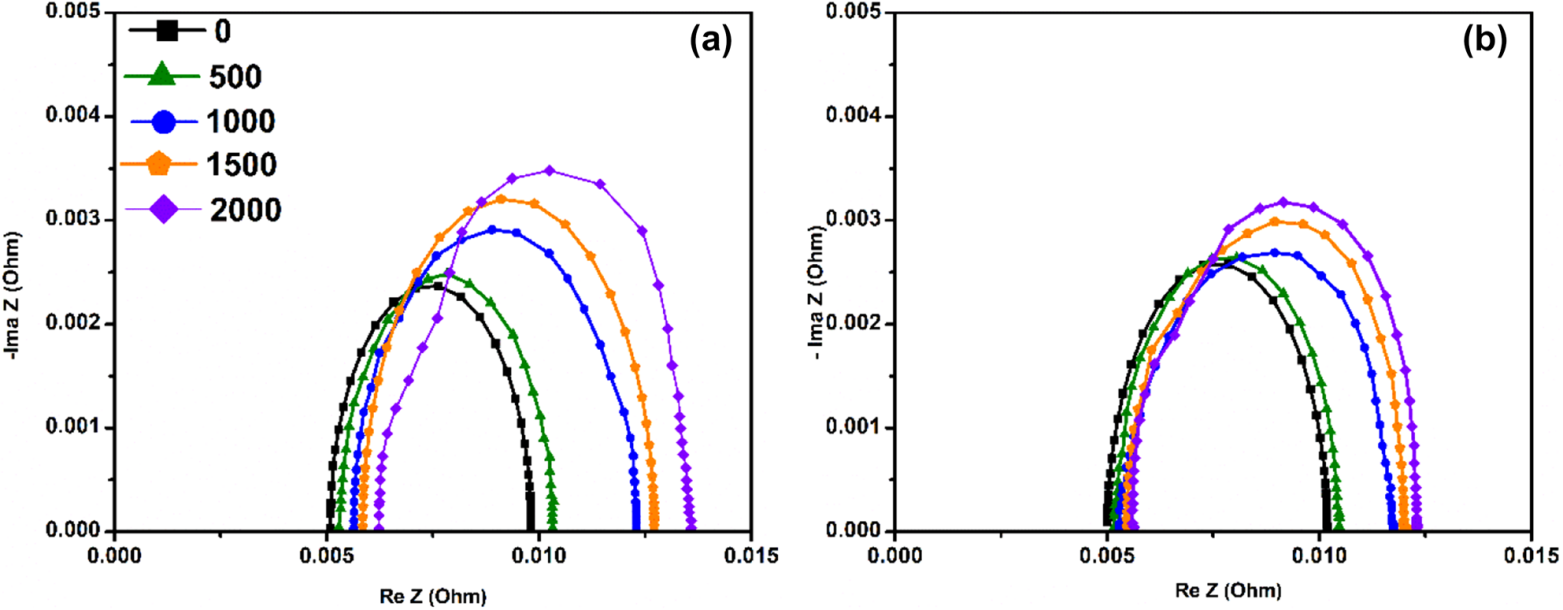
Nyquist plot of (**a**) DLC1 and (**b**) DLC2 for 0, 500, 1000, 1500, and 2000th cycles.

EIS is a non-destructive in-situ investigation of the structural and material properties of a fuel cell and is also an important tool in evaluating the degradation behaviour of fuel cells subjected to the durability testing. The ohmic resistance of a fuel cell is the total sum of resistance offered by the cell components and their contact resistance. Here, the high-frequency region represents the ohmic resistance. Membrane conductivity is one of the major contributors to ohmic resistance, which is influenced by the intricate relationship between current density, water content, and thermal management. EIS curves were measured at 0.5 V for every 100 cycles and Fig. 5, represents the Nyquist plot for DLC1 and DLC2. Using EC-Lab software, the Nyquist curves were calculated.

From Fig. 5, the common observation was that the ohmic resistance keeps increasing, and there was a significant increase in the diameter of the semicircle for both dynamic load conditions after 1000 cycles. Increase in semicircle diameter implies that the charge transfer resistance increases. It can be clearly seen that the DLC1 semicircle diameter was significantly higher than the diameter of DLC2’s semicircle. This is a clear indication that the catalyst layer of DLC1 was more severely damaged than DLC2.

Another inference that can be made from Fig. 5, is the increase in the ohmic resistance of the cells. In DLC1, the initial resistance value was 5.08 mΩ which increased to 5.82 mΩ, accounting for a 14.56% increase, while the cell operated under DLC2 had an initial resistance of 5.01 mΩ, which increased to 5.52 mΩ at the end of 2000 cycles. This accounts for a 10.18% increase in total resistance. From EIS analysis, it’s clear that a higher value of charge transfer resistance denoted the sluggish kinetic of the ORR. This also indicates that there was severe loss of catalyst active area during both high and low current density operation in DLC1 compared to DLC2.

### MEA cross-section FESEM analysis after 2000 dynamic load cycles

**Figure 6:**
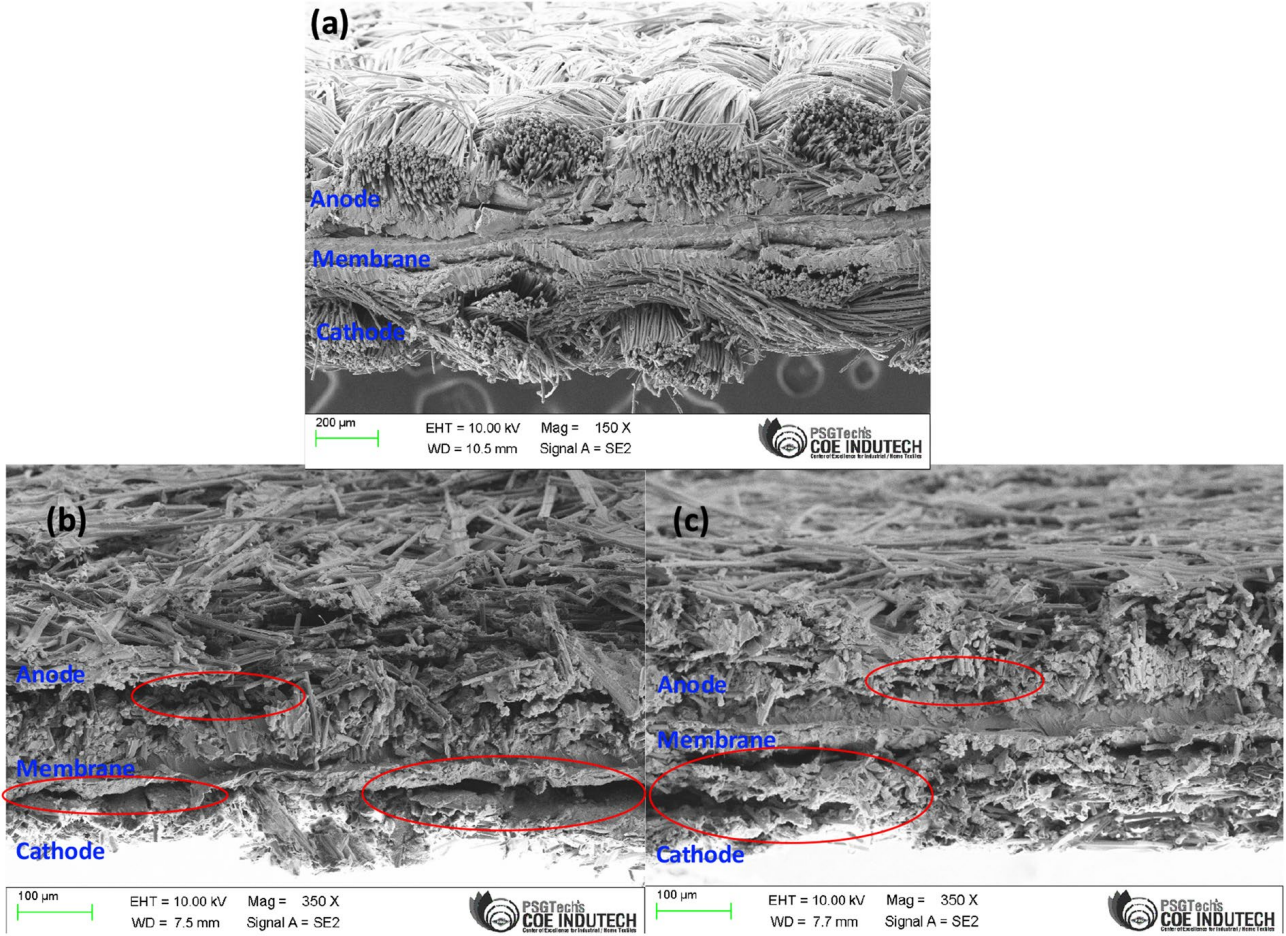
Cross-section FESEM image of (**a**) Fresh MEA (**b**) DLC1 and (**c**) DLC2.

The catalyst layer morphology was analysed using FESEM imaging. Figure 6, shows the cross-sectional FESEM image of both MEAs used in DLC1 and DLC2. It can be observed that there are changes in the catalyst layer, especially on the cathode side of the MEA. This effect is more severe in DLC1. Another observation is the delamination of catalyst and membrane, which adversely affect the performance of the fuel cells. These delaminated areas are circled in red in Fig. 6.

The reason for these morphological changes might be the carbon corrosion occurring due to high voltage when operating under low current density and flooding due to high current density^48^. Cell reversal is another factor that leads to carbon corrosion, during the load change there is short-time reactant starvation occurring at the catalyst site at the anode. This increases the anode potential and leads to carbon corrosion^49,50^. In general, the higher the ramp rate, the higher will be the catalyst layer degradation, this is the reason for the severe degradation of DLC1’s MEA.

## Conclusion

In this work, the effect of ramp rate on performance degradation of PEMFC is deliberated, and the factors that influence the degradation rates are elucidated. Two similar fuel cells were subjected to different dynamic loading conditions with different ramp rates. The performance degradation of the fuel cells can be evaluated from the voltage decay rate. The following conclusions were made:

1. It can be concluded that the performance degradation is closely related to the ramp rate of the loading cycle and as the number of dynamic load cycles increases, the performance degradation is accelerated. Analysis of polarization curves, EIS, and FESEM images gives a clear indication of performance degradation. DLC2 had a lower ramp rate and the voltage decay observed was 10 µV/cycle, while in DLC1 the voltage degradation observed was 19 µV/cycle.
2. The combined performance degradation of DLC1 and DLC2 was 20.581% and 10.78% after 2000 cycles. The highest performance drop of DLC1 was due to the higher current ramp rate. This shows the performance degradation was highly influenced by the ramp rate.
3. Another important observation was the increase of ohmic resistance, the increase was higher in DLC1 and comparatively lower in DLC2. This is due to the delamination effect observed in the MEA. 14.56% increase in ohmic resistance was observed in DLC1 which is higher than DLC1. Delamination effect is clearly visible in Fig. 6.
4. The main reasons for the performance degradation are the short-term reactant starvation during load change, high potential operation, and high current density operation. All these factors lead to carbon corrosion in various degrees in both anode and cathode altering the structure of the catalyst layer. This phenomenon causes irreversible damage to the MEA that reduces the useful operating time of the fuel cell.
5. The performance degradation can be extenuated by reducing the ramp rate. The effect can be clearly seen in DLC2 which has a lower voltage decay rate than DLC1. To mitigate the effect of reactant starvation, excess reactant can be supplied if the conditions permit and the effect of flooding in high current density operation can be attenuated by the formulation of an effective purging strategy for effective water management. Purging has many other positive effects on the performance of the fuel cell in addition to water removal, purging strategy will be formulated and optimized in future studies.

## Data Availability

Datasets are available from the corresponding authors on reasonable request.
